# Florid Mesothelial Hyperplasia Associated with Abdominal Wall Endometriosis Mimicking Invasive Carcinoma

**DOI:** 10.1155/2021/3439700

**Published:** 2021-11-28

**Authors:** Edgar G. Fischer, Shweta Agarwal

**Affiliations:** Division of Surgical Pathology and Cytopathology, Department of Pathology MSC08 4640, 1 University of New Mexico, Albuquerque, NM 87131, USA

## Abstract

Florid mesothelial hyperplasia typically occurs in the pelvis, abdomen, or chest associated with an underlying neoplastic or inflammatory process. These lesions are of clinical significance because they can mimic a neoplasm. Early reports were published in the 1970s, but only a few case series of such lesions have been published in the gynecologic pathology literature. Here, we report a case of florid mesothelial hyperplasia with an infiltrative growth pattern, mimicking an invasive carcinoma. The lesion was associated with endometriosis forming a mass lesion in the abdominal wall. Histologically, tubular arrangements and nests of mesothelial cells, some with artifactual slit-like spaces, formed a stellate lesion adjacent to endometrial glands and stroma. Cytologic atypia was mild and reactive, and positive immunostaining for calretinin, WT-1, and cytokeratin 5 identified the lesion as mesothelial and benign. We describe in detail the histologic findings in this case and review the pertinent literature. We discuss the clinically importance of this diagnostic pitfall and the path to arriving at the correct diagnosis.

## 1. Introduction

Mesothelial hyperplasia most commonly occurs in the pelvic and abdominal cavity as a reactive process, typically in response to an underlying neoplastic or inflammatory process. Florid lesions of mesothelial hyperplasia are clinically significant when they become a diagnostic challenge and need to be distinguished from a neoplastic process. Early descriptions of mesothelial hyperplasia arising in hernia sacs go back to the 1970s [1, 2], but the number of published case series and case reports remains relatively small [1–5]. The pleural [6, 7] and pericardial cavity as well as the tunical vaginalis of the testis [8, 9] can also be affected. Our review of the gynecologic pathology literature found 55 cases of florid mesothelial hyperplasia described in three case series [3–5]. Florid mesothelial hyperplasia can mimic a malignant neoplasm in patients with otherwise benign lesions or with borderline ovarian tumors. When found in staging biopsies in patients with pelvic or abdominal tumors, misinterpretation of these lesions as tumor deposits can potentially lead to erroneous upstaging with significant clinical implications [3, 5, 10, 11]. In the vast majority of cases, florid mesothelial hyperplasia arises as papillary or nodular excrescences associated with a mesothelium-lined surface. The case presented here is different and unusual, because the hyperplastic lesion was entrapped in fibrous tissue associated with a solid mass created by endometriosis in the abdominal wall. In this report, we describe in detail the histologic findings and the immunohistochemical workup that lead to the correct diagnosis and summarize the pertinent pathology literature.

## 2. Report of a Case

A 32-year-old female with a history of prior cesarean section presented with endometriosis forming an abdominal wall mass at the Pfannenstiel incision site. She was on progestin treatment for her endometriosis. A 4.6 cm greatest diameter portion of abdominal wall soft tissue was excised and revealed a 2.6 cm greatest diameter ill-defined firm mass lesion. The cut surface was yellow-tan with punctate areas of red-brown discoloration.

**Figure 1:**
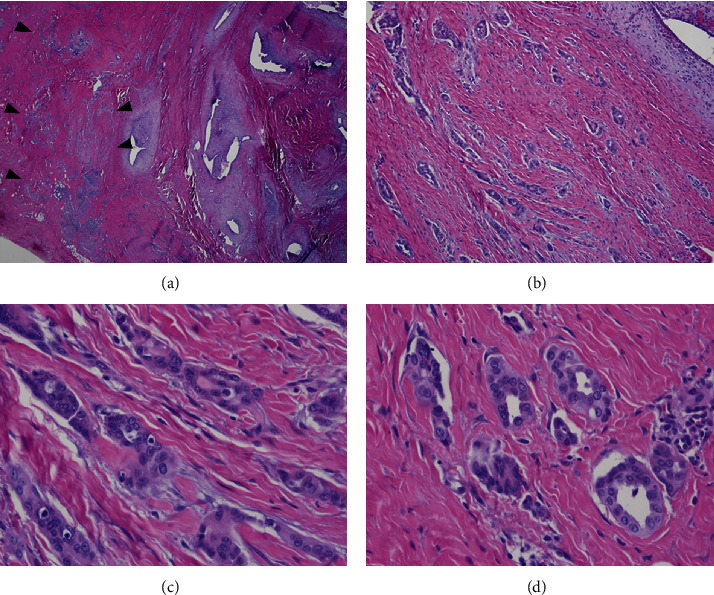
Scanning magnification shows groups of endometriotic glands surrounded by light gray staining stroma that is pseudodecidualized due to progestin treatment. Adjacent is a focus of tubular structures and nests with a pseudoinfiltrative growth pattern (arrowheads) (a, 20x). At intermediate magnification cells form tubular structures and nests with angular pseudoinfiltrative shapes, some surrounded by slit-like spaces set in dense collagenous stroma. The endometriotic gland in the upper right corner is surrounded by pseudodecidualized stroma (b, 100x). High magnification reveals the pseudoinfiltrative growth pattern of tubular structures and nests. Cells have relatively scant eosinophilic cytoplasm and round to oval monotonous nuclei, with some nuclear overlap. Chromatin is finely distributed, and occasional small nucleoli are seen (c, d; 400x).

**Figure 2:**
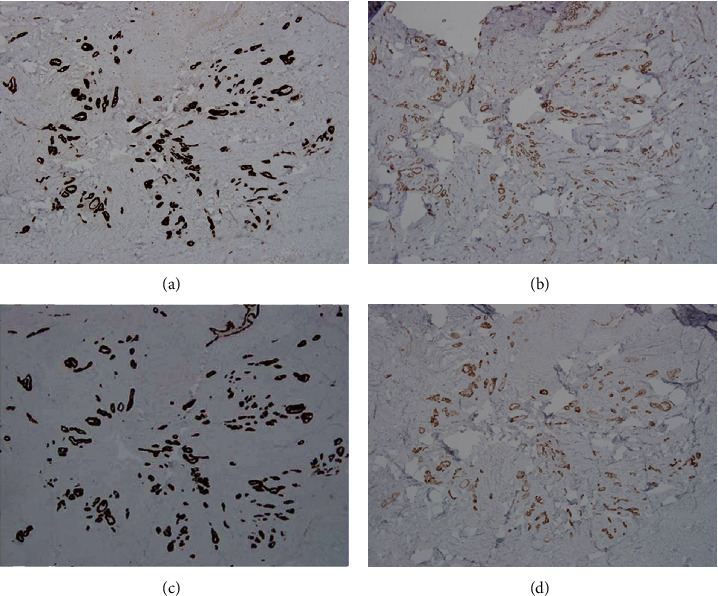
Immunohistochemical stains for the mesothelial markers calretinin (a) and WT-1 (b) are strongly positive. Cytokeratin 7 (c) and cytokeratin 5 (d) are also positive (all 40x). These immunostains highlight the pseudoinfiltrative architecture at low-magnification images.

Histologically, the mass lesion was predominated by groups of endometrial glands and stroma. The endometrial stroma appeared light gray due to pseudodecidual change caused by progestin treatment (Figure 1). Adjacent to the endometriotic glands was a 5 mm focus of tubular structures and nests with a pseudoinfiltrative growth pattern (Figure 1(a) arrowheads). Intermediate magnification revealed tubular structures and nests with angulated pseudoinfiltrative shapes; some surrounded by artifactual slit-like retraction spaces, in a background of dense fibrosis. At high magnification, cells had relatively scant eosinophilic cytoplasm. Nuclei were relatively monotonous with round to oval shapes and some nuclear overlap. Chromatin was finely distributed, and occasional small nucleoli were seen. Immunohistochemical stains revealed the lesional cell to be positive for the mesothelial markers calretinin and WT-1 and also positive for cytokeratins 7 and 5 (Figure 2). These immunostains particularly highlight the stellate and pseudoinfiltrative growth pattern. Immunostains for estrogen receptor, GATA-3, TTF-1, and CD10 were negative. There was weak nonspecific staining for Pax-8.

## 3. Discussion

Our case of mesothelial hyperplasia is a rare example of a florid reactive process with an infiltrative appearance that can present a diagnostic challenge to the surgical pathologist. The stellate shape, highlighted by the immunopstains, and the infiltrative pattern mimicked an invasive carcinoma. The lesion was set in an area of dense fibrosis, was part of a mass-forming lesion, and was positive for cytokeratins by immunohistochemistry. Important clues to the correct diagnosis and to avoiding misinterpretation as a carcinoma were the small size of the lesion, its association with a prior abdominal wall surgical site and with endometriosis, and absence of a history of malignancy. Despite the infiltrative growth pattern, the degree of cytologic atypia was mild, but accentuated by high magnification. Cells were small with relatively small nuclei, even chromatin distribution, and only small nucleoli. By contrast, adenocarcinomas arising in association with endometriosis typically have the histology of either endometrioid adenocarcinoma or clear cell carcinoma. Finally, the immunoprofile resolved the diagnostic challenge, with positive staining for the mesothelial markers calretinin, WT-1, and cytokeratin 5.

While our lesion was located in an area of dense fibrosis, most cases of florid mesothelial hyperplasia are associated with a mesothelial surface, with a piling up of proliferating cells from the surface lining and prominent reactive cytologic atypia. Mesothelial hyperplasia is typically associated with another underlying pathology such as endometriosis, an inflammatory process, or a neoplasm. In our case, the lesion most likely arose from mesothelial cells that were entrapped in the abdominal wall connective tissue during a prior cesarean section.

The earliest descriptions of mesothelial hyperplasia mimicking a neoplasm in hernia sacs were published in the 1970s [1, 2]. Our literature review found 55 cases of florid mesothelial hyperplasia in three case series described in the gynecologic pathology literature [3–5]. In 1981, Kerner et al. described unusual mesothelial inclusions in the ovary, fallopian tube, and the pelvic wall of six cases, who were part of a cohort of 57 women with endometriosis. The authors did not find any such lesions in 100 ovaries from a control group of patients without endometriosis [4]. They described these mesothelial inclusions as small closely packed nests of mesothelial cells with or without central lumina. Some of these lesions had a pseudoinfiltrative growth pattern, highlighting the potential diagnostic challenge. All cases of mesothelial hyperplasia in this series occurred in patients with endometriosis, and the authors suggested an association with a common stimulus responsible for their development [4]. In 1993, Clement and Young published five cases of florid mesothelial hyperplasia associated with ovarian tumors. In four of these cases, the mesothelial lesions initially created diagnostic difficulty in terms of their histologic classification and the staging of the associated tumors [3]. In two cases, the mesothelial proliferations occurred in the cyst wall of one serous and one mucinous borderline tumor and mimicked an invasive carcinoma. In the other three cases, the lesions occurred in the extraovarian pelvic peritoneum and mimicked a metastatic lesion [3].

In the largest series to date, Oparka et al. reported 44 cases of florid mesothelial hyperplasia that presented diagnostic difficulties, most of them occurring on the surface of the ovary [5]. In 38 cases, these mesothelial lesions were associated with nonneoplastic processes, most commonly ovarian endometriosis, but also tuboovarian abscesses, paraovarian cysts, follicular cysts, and in one case with ovarian torsion. Histologically, most cases in this series featured small tubules, some dilated, as well as solid nests and cords of cells. In many cases, the lesions were characteristically embedded in abundant fibrous stroma, often resulting in a linear pattern of tubules, i.e., nests and cords arranged parallel to the ovarian surface. Retraction spaces around cell nests simulated vascular invasion. Occasionally, groups of cells appeared to be present in true vascular spaces, which in one case were illustrated with a positive CD34 immunostain [5]. Some cases had a minor component with papillary architecture. Cytologically, lesions cells had central, regular, round, and often vesicular nuclei. Nuclear atypia was mild, with only very occasional mitoses. The cytoplasm was eosinophilic and varied from scant to relatively abundant. A second architectural feature of closely packed small glands and papillae was present in a minority of cases, with similar cytologic features. These lesions were significant because they mimicked a serous proliferation, and one case even had psammoma bodies [5]. These findings highlight the importance of distinguishing mesothelial lesions from a serous neoplasm, either from invasion in an ovarian tumor or from tumor involvement at an extraovarian peritoneal site. Immunohistochemical stains were performed in 19 cases, with calretinin as the most commonly positive marker, and the epithelial marker BerEP4 as the most commonly negative stain. The authors concluded that calretinin and BerEp4 were the most sensitive and specific markers. Other commonly used markers for mesothelial differentiation include podoplanin (D2-40), thrombomodulin, WT-1, and cytokeratin 5/6. Commonly used markers for epithelial glandular differentiation include MOC-31, B72.3, CEA, and CD15 [5]. Pax-8 and estrogen receptor are often used as markers for epithelial tumors of Müllerian origin.

In conclusion, we present a case of florid mesothelial hyperplasia with an infiltrative growth pattern, mimicking a carcinoma. The lesion was associated with abdominal wall endometriosis forming a mass lesion. In such cases, detailed attention to histologic findings and a selective panel of immunohistochemical stains will lead to the correct diagnosis.

## Data Availability

No supporting materials such as data, new software, databases, or raw data were used for this report.
